# ZnO/SiO_2_ Filler-Incorporated Resin Composites for Vat Photopolymerization of Dental Restorations with Antimicrobial Efficacy

**DOI:** 10.3390/ma18163909

**Published:** 2025-08-21

**Authors:** Jong-Won Jeon, Gyu-Nam Kim, Jae-Min Jung, Young-Hag Koh

**Affiliations:** 1 School of Biomedical Engineering, Korea University, Seoul 02841, Republic of Korea; rnxh125@naver.com (J.-W.J.); gyunamkd@korea.ac.kr (G.-N.K.); optima3000@daum.net (J.-M.J.); 2 Interdisciplinary Program in Precision Public Health, Korea University, Seoul 02841, Republic of Korea

**Keywords:** ceramic–resin composite, ZnO/SiO_2_ ceramic filler, antimicrobial effect, provisional dental restoration, LCD-3D printing

## Abstract

This study aimed to develop dental resin composites containing ZnO/SiO_2_ ceramic particles as an antimicrobial filler for producing provisional dental restorations using the lithography-based liquid crystal display (LCD) 3D printing technique. Three types of dental resin-ceramic composites with different filler contents (0 wt%, 5 wt%, and 10 wt%) were prepared to offer high antimicrobial efficacy. Printing parameters, particularly off-time, were optimized for each composition to achieve high-quality prints. Mechanical testing demonstrated increased hardness and elastic modulus. In addition, the 10 vol% composite exhibited a three-point flexural strength of 113.4 MPa, exceeding the 100 MPa requirement specified in ISO 4049:2019 for provisional dental materials. Antimicrobial testing showed a significant reduction in *Streptococcus mutans* colonies, with up to 84.4% decrease for the 10 vol% composite compared to the unfilled resin. A provisional 3-unit bridge was successfully printed using the 10 vol% composite, demonstrating practical applicability.

## 1. Introduction

Additive manufacturing (AM) technologies have revolutionized the field of dentistry, offering new possibilities for the fabrication of provisional restorations. These interim dental prostheses play a crucial role in evaluating phonetic and occlusal functions prior to permanent implant placement while preserving and enhancing peri-implant and gingival tissues [1,2]. AM techniques, particularly 3D printing, allow for rapid production of customized provisional restorations with high precision and reproducibility [3,4]. This approach not only streamlines the manufacturing process but also enables the incorporation of advanced materials to enhance the functional properties of these temporary dental devices [5,6,7,8,9,10].

Among various AM techniques, lithography-based liquid crystal display (LCD) 3D printing has emerged as a promising method for producing high-resolution dental restorations [11,12]. This technology utilizes an LCD screen to project light patterns onto a photosensitive resin, curing entire layers simultaneously. This LCD-based approach offers several advantages over traditional stereolithography (SLA) or digital light processing (DLP) methods, including lower cost, reduced maintenance, and the potential for larger build volumes [13,14]. The high-resolution LCD screens allow for precise control over pixel size, resulting in excellent feature detail and smooth surface finishes, which are critical for dental applications [15,16]. Additionally, the use of LCD technology enables faster print speeds and more uniform light distribution across the build area, leading to improved consistency of the final printed parts [17,18].

Zinc oxide–silica (ZnO/SiO_2_) ceramic fillers have gained significant attention in the biomedical field due to their unique properties [19,20,21]. ZnO is well-known for its antimicrobial activity, which can help prevent bacterial colonization on dental surfaces [22]. Additionally, the incorporation of SiO_2_ can enhance mechanical properties and biocompatibility of composite materials [14,23]. The synergistic effects of ZnO and SiO_2_ in ceramic fillers offer potential benefits for dental applications, including improved strength, wear resistance, and antimicrobial activity [19,24]. Integration of ZnO/SiO_2_ ceramic fillers into photocurable resins for lithography-based LCD 3D printing presents an opportunity to develop advanced materials for provisional dental restorations [19,24,25]. By carefully controlling the composition and distribution of these ceramic particles within the resin matrix, it is possible to tailor the mechanical, optical, and antimicrobial properties of printed structures [25,26,27,28,29]. However, the incorporation of ceramic fillers can also affect the curing behavior and printability of resin composites. Thus, careful optimization of material formulation and printing parameters is required [30,31,32,33,34,35].

**Figure 1:**
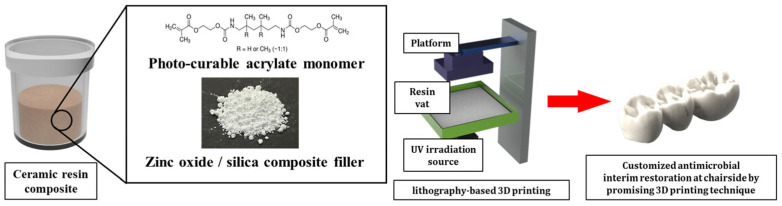
Schematic diagram of antimicrobial resin-ceramic composites with ZnO/SiO_2_ ceramic filler for DLP of provisional crown and bridge.

This work aimed to develop and characterize resin composites (dental resin–ZnO/SiO_2_ ceramic fillers) for lithography-based LCD 3D printing of provisional dental restorations (Figure 1). In this work, the effects of different ZnO/SiO_2_ filler concentrations on curing behavior, mechanical properties, and antimicrobial activity of printed structures were comprehensively evaluated. To guide this investigation, the null hypothesis was that the incorporation of ZnO/SiO_2_ ceramic fillers into the dental resin matrix would not significantly affect the mechanical or antimicrobial properties of the printed composite. Furthermore, LCD printing parameters were optimized to achieve high-quality provisional restorations with enhanced functionality. By combining the advantages of lithography-based LCD 3D printing with the unique properties of ZnO/SiO_2_ ceramic fillers, this work seeks to advance the field of digital dentistry and provide improved solutions for temporary dental prostheses.

## 2. Materials and Methods

### 2.1. Starting Materials and Preparation of ZnO/SiO_2_ Resin-Ceramic Composite Suspension

Constituents of the antimicrobial ZnO/SiO_2_ resin-ceramic composite suspension for fabricating provisional crown and bridge are summarized in Table 1. Commercially available Zinc oxide/silica ceramic filler (ZnO/SiO_2_, Sukgyung AT, Gyeonggi-do, Republic of Korea) was used for antimicrobial effects.

To formulate the photocurable resin matrix suitable for LCD-based vat photopolymerization, a ternary monomer system consisting of diurethane dimethacrylate (UDMA; Sigma Aldrich, St. Louis, MO, USA), triethylene glycol dimethacrylate (TEGDMA; Tokyo Chemical Industry, Tokyo, Japan), and 2-hydroxyethyl methacrylate (HEMA; Sigma Aldrich, St. Louis, MO, USA) was employed. UDMA was chosen as the primary base monomer (50 wt%) due to its high mechanical strength, low polymerization shrinkage, and biocompatibility [4]. TEGDMA (20 wt%) was added to further enhance the crosslinking density of the polymer network, thereby improving mechanical properties and curing efficiency. HEMA (30 wt%) was used as a reactive diluent to reduce the overall viscosity of the resin, thereby promoting the dispersion of ceramic fillers, and to reduce the polymerization shrinkage of resin-ceramic composite [36].

The final formulation of 50:20:30 (UDMA/TEGDMA/HEMA, wt%) was optimized through preliminary evaluations to balance low viscosity, filler dispersion, and mechanical performance, while maintaining compatibility with LCD-based 3D printing. This formulation allowed stable dispersion of ZnO/SiO_2_ ceramic fillers within the resin, resulting in printable suspensions for LCD 3D printing with desirable flow and curing characteristics. Furthermore, DISPERBYK-2001 (BYK-Chemie GmbH, Wesel, Germany) was used as a dispersant for uniform filler distribution in the resin composite. Diphenyl (2,4,6- trimethylbenzoyl) phosphine oxide (TPO; Sigma Aldrich, St. Louis, MO, USA) was used as a photoinitiator.

Within the resin-ceramic composite suspension, different amounts (0 vol%, 5 vol%, and 10 vol% to total amount of suspension) of ZnO/SiO_2_ ceramic fillers were introduced into the UDMA/TEGDMA/HEMA blend, along with the dispersant (3 wt% relative to the ZnO/SiO_2_ filler content). The photoinitiator (3 wt% relative to the total monomer content) was then added and mixed vigorously using a planetary centrifugal mixer (Hantech Co, Ltd., Gyeonggi-do, Korea) for 40 min at 1000 rpm. The mixture was later used for LCD 3D printing.

### 2.2. Characterization of ZnO/SiO_2_ Filler

Energy-dispersive X-ray spectroscopy (EDS; Horiba, Ltd., Kyoto, Japan) attached to a scanning electron microscope (SEM; JSM-6701F, JEOL Techniques, Tokyo, Japan) was used to analyze the elemental composition of the ZnO/SiO_2_ ceramic filler. Note that for SEM observations, surfaces of powders and printed samples were coated with gold using a magnetron sputter coater (108Auto, Cressington, Watford, UK) at 20 mA for 120 s under high vacuum and then SEM images were taken at an acceleration voltage of 10 kV with a working distance of 8 mm. EDS point analyses were performed at ×30,000 magnification with a spot size of 0.4 μm to evaluate the weight percentages of Zn, Si, and O atoms. A total of 5 points were analyzed across ZnO/SiO_2_ ceramic fillers to ensure representative measurements. Weight percentages (%) of Zn, Si, and O atoms were then measured, excluding other atoms, which were calculated to weight percentages of ZnO and SiO_2_ within the ZnO/SiO_2_ filler.

X-ray diffraction (XRD) analysis of the raw ZnO/SiO_2_ powder was performed using a Rigaku Miniflex II diffractometer (Rigaku, Tokyo, Japan) with Cu Kα radiation (anode material: copper, λ = 1.5406 Å) to identify the crystalline phases of ZnO and SiO_2_. The measurement was conducted over a 2*θ* scan range of 5° to 80° with a step size of 0.02°/step, a scan speed of 2°/min, and operating conditions of 40 kV and 40 mA. Particle-size distribution of the ZnO/SiO_2_ filler was characterized using a dynamic light scattering method (DLS; Zetasizer Nano ZS90, Westborough, MA, USA). Morphologies and sizes of the primary and secondary particles were examined using scanning electron microscopy (FE-SEM; JSM-6701F, JEOL Techniques, Tokyo, Japan). The ability of the ZnO/SiO_2_ ceramic filler to generate reactive oxygen species (ROS) was evaluated using a nitroblue tetrazolium (NBT) assay. A 20 mL NBT solution (0.15 mM, distilled water solvent) was mixed with 1.0 g of ZnO/SiO_2_ filler, and an LED light in the laboratory environment was used to irradiate the mixed solution with different irradiation durations (0, 30, 60, and 120 min). The solution was measured using a UV-Vis spectrophotometer under the following conditions: a scan range of 200–500 nm (absorbance peak of NBT: 259 nm), a scan speed of 600 nm/min, and a base solvent of distilled water. The absorbance spectrum of the NBT solution was measured by UV-Vis spectroscopy with or without ZnO/SiO_2_ filler under LED light (wavelength of ~400–700 nm) for 30, 60, and 120 min.

### 2.3. Characterization of ZnO/SiO_2_ Resin Composite Suspension

Rheological behaviors of resin composites with 10 vol% of ZnO/SiO_2_ filler and different dispersant concentrations (1, 2, 3, and 4 wt%) were measured using a cone/plate rheometer (DV3T-HB Rheometer, Brookfield Inc., Middleboro, MA, USA). Measurements were taken over a shear rate range from 0.6 s^−1^ to 150.0 s^−1^ to optimize the amount of dispersant. Additionally, resin composites with different ZnO/SiO_2_ filler contents (0, 5, and 10 vol%) were evaluated. Photo-curing behavior of resin-ceramic composites with different ZnO/SiO_2_ filler contents (0, 5, and 10 vol%) was characterized to determine the optimal photo-curing time for the resin-ceramic composite suspension. All resin-ceramic composite suspensions with different filler contents were exposed to a UV light (intensity of ~0.66 mW/cm^2^) to induce photopolymerization for various exposure times. Subsequently, thicknesses of photopolymerized layers were measured using vernier calipers.

To further analyze the photo-curing behavior of the resin suspensions, differential scanning calorimetry (DSC 4000, Perkin Elmer, Waltham, MA, USA) equipped with a UV irradiation source (Omnicure Series 2000, Excelitas, Billerica, MA, USA) was used under isothermal conditions at 25 °C. A UV light source (rather than a clinical LED unit) was selected to ensure precise control of irradiation parameters and consistent energy output, which are critical for obtaining reproducible curing data across all samples. The resin composite samples were exposed to UV light at a power of approximately 0.6676 mW/cm^2^ for 120 s, which was not intended to simulate clinical curing conditions, but rather to capture the full extent of the polymerization reaction and determine the total reaction enthalpy (Δ*H_t_* (J/g)) and degree of conversion. This duration ensured stabilization of the heat flow signal and integration of the complete polymerization process. The heat flow generated during curing was recorded and used to calculate total enthalpy by integrating the area under the curve relative to the stabilized baseline at 120 s. Monomer reaction enthalpy (Δ*H_m_* (J/g)) and percent conversion (*a(t)*) were calculated using the following equations [37]:

Δ*H_m_* = Δ*H_t_* ∗ *(wt% of monomer in total resin amount)* (1)

(2) $$Percent\;conversion\;a(t)\;[\%]=\left[\frac{∆H\left(t\right)}{n\times ∆H_{0}\times m}\right]\times 100\;[\%]$$

where Δ*H(t)* is the total heat evolved at time *t*, *n* is the number of carbon double bonds per monomer, Δ*H*_0_ is the standard enthalpy of reaction for the acrylate double bond (86.2 kJ/mol), and *m* is moles of acrylate monomer used in this work. To ensure precise 3D printing of resin composites with different viscosities, 3D printing conditions were optimized by controlling the off-time, which is the delay time that the printing platform waited within the printing vat with resin composite suspension before curing a single layer. The off-time was controlled from 0 to 90 s with an interval of 30 s. The optimal off-time condition was determined by printing nine square-shaped specimens with dimensions of 30 × 30 × 2 mm, followed by measuring the sizes of the printed samples at each condition.

To fabricate test specimens for mechanical and antimicrobial evaluation, a lithography-based LCD 3D printer (Anycubic Photon Mono 6K, Anycubic, Shenzhen, China) was employed. The printer is equipped with a monochrome LCD panel of 6K resolution (5760 × 3600 pixels), offering a pixel size of approximately 34.4 μm, which provides high-resolution layer-by-layer fabrication suitable for dental applications. The thickness of each printed layer was fixed at 50 μm, and the curing time for each layer was set to 2.5 s based on preliminary curing depth experiments to ensure complete polymerization of each 50 μm layer. The average light intensity of the printer’s UV source was ~0.66 mW/cm^2^, with a wavelength of ~405 nm, which closely matched the intensity used in the photo-DSC experiments. To accommodate varying viscosities of suspensions with different ZnO/SiO_2_ filler contents, an off-time (i.e., the delay period before exposure after recoating) ranging from 30 to 90 s was applied and optimized for each formulation. These optimized printing conditions enabled the successful fabrication of test specimens and demonstration structures, including the 3-unit provisional dental bridge, with high dimensional accuracy and surface quality.

### 2.4. Evaluation of ZnO/SiO_2_ Filler Distribution in Resin-Ceramic Composites

Distribution of ZnO/SiO_2_ filler within the printed resin composites was evaluated using multiple characterization techniques. TGA analysis was conducted to evaluate the weight percent of ZnO/SiO_2_ in the resin-ceramic composites by measuring the weight loss percent [%]. All samples were thoroughly polished before any measurements were taken. These samples were heated up to 585 °C at a heating rate of 10 °C/min in air. Their weight losses over temperature were then recorded. Furthermore, the filler distribution was characterized by image analysis using SEM, which was performed for resin-ceramic composites with different filler contents (0, 5, and 10 vol%). The fractured surface of each specimen was magnified using a ×10,000 magnification at 10 keV. These analyses were performed to roughly assess the uniformity of filler distribution throughout the printed resin-ceramic composite and to identify any potential agglomeration or sedimentation of ZnO/SiO_2_ particles within the resin matrix.

### 2.5. Evaluation of Mechanical Properties of Resin-Ceramic Composites

Mechanical properties of resin-ceramic composites with different filler contents were evaluated using three-point flexural strength tests in accordance with ISO 4049:2019 and the Vickers hardness test [38]. All specimens used for evaluating mechanical properties were carefully polished before any testing. Three-point flexural strength tests were performed by applying the load to a rod placed at the center of specimens, which were supported by two rods with a distance of 20 mm (*L*) at a constant crosshead speed of 1 mm/min, using a universal testing machine (UTM; ST-1001, SALT, Incheon, Republic of Korea). Dimensions of all bar-shaped specimens were 25.0 mm, 2.0 mm, and 2.0 mm for length (*l*), width (*b*), and thickness (*h*), respectively, which were measured using a micrometer. The flexural strength (*σ*) and modulus (*E*) of tested specimens (*n* = 10 for each filler content) were calculated by considering the load (*F_f_*) using the following equation [38,39]:

(3) σ = 3· Ff·L2·b·h2

(4) E=L3·m4·b·d3

where *F_f_* is the load at fracture [*N*], *L* is the distance between the supporting rods [mm], and *b* and *h* are the width and thickness of bar-shaped specimens [mm]. Note that *m* is the slope of the modulus line (N/mm), which was calculated from the slope of the linear portion of the stress–strain curve.

Hardness values of resin composites with different filler contents were measured using a Vickers Hardness Testing Machine (HV-100 Series, Mitutoyo, Kawasaki, Japan) with a load of 294.2 N, following the method outlined in the ISO 6507-1 standard [40]. Dimensions of all disk-shaped specimens were 20.0 mm and 3.5 mm for diameter and height, respectively. The Vickers hardness (*H_v_*) of tested specimens (*n* = 10 for each filler content) was calculated using the following equation:

(5) Hv = 0.1891×F(d1×d2)

where *F* is the applied force for the hardness test [N], and *d*_1_ and *d*_2_ are the lengths of the diagonal indentation [mm]. Mechanical tests were performed to assess the impacts of ZnO/SiO_2_ filler content on hardness, strength, and elastic properties of the printed resin-ceramic composites.

### 2.6. Evaluation of Antimicrobial Effects of Resin-Ceramic Composites

Antimicrobial activities of ZnO/SiO_2_ resin composites were evaluated using *Streptococcus mutans* (*S. mutans*) cultured in LB (Luria-Bertani) broth. Printed disk specimens with a diameter of 12.0 mm and a height of 2.0 mm were prepared. They were thoroughly polished before evaluation. A minimum of five specimens were tested for each composition. The colony-forming unit (CFU) method was employed to evaluate antimicrobial activity over a four-day period. On the first day, *S. mutans* (50 μL) from the bacteria stock was vortexed with 3.0 mL of LB broth and cultured at 37 °C for 24 h at 200 rpm. Test specimens were also placed in a 24-well plate and sterilized using a UV lamp. On the second day, the new LB broth was mixed with the cultured *S. mutans* at a 3:1 ratio. This bacterial suspension was then added to a 24-well plate containing test specimens and cultured for an additional 24 h at 37 °C. The third day involved washing cultured specimens three times with 1 mL of DPBS (Dulbecco’s Phosphate-Buffered Saline) to remove non-adherent bacteria. Washed specimens were then added to 3 mL of DPBS, and 100 μL of this suspension was further diluted using 1 mL of DPBS. The diluted suspension was subsequently spread onto LB agar plates. On the final day, after 24 h of incubation, colonies cultured on LB agar plates were counted. Throughout the experiment, culturing conditions were maintained under LED light (400–700 nm) in the laboratory at 37 °C. The antimicrobial activity was quantified by comparing numbers of viable bacteria (CFU count) on ZnO/SiO_2_-containing specimens with those on control specimens (0 vol% ZnO/SiO_2_). This method allows for assessment of the antimicrobial efficacy of ZnO/SiO_2_ resin composites against *S. mutans*, a common oral pathogen associated with dental caries.

### 2.7. Statistical Analysis

Before performing all parametric statistics, the normality of distributions was verified with the Shapiro–Wilk test. All data are expressed as mean ± standard deviation. All statistical analyses were performed using one-way analysis of variance (ANOVA) with Tukey’s post-hoc test in MATLAB (Release 2024b, The MathWorks, Inc., Natick, MA, USA). A *p*-value < 0.05 was considered statistically significant and is graphically demonstrated with asterisks in each figure.

## 3. Results and Discussion

### 3.1. Characteristics of ZnO/SiO_2_ Filler

**Figure 2:**
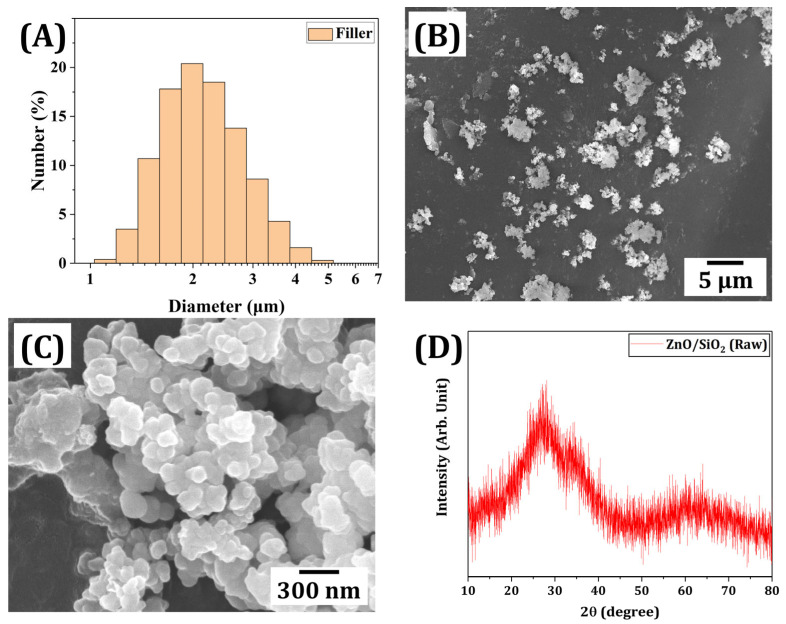
(**A**) Size distributions of ZnO/SiO_2_ particles. Representative FE-SEM images showing (**B**) secondary, (**C**) primary ZnO/SiO_2_ particles, and (**D**) XRD patterns of ZnO/SiO_2_ particles used in this work.

The ZnO/SiO_2_ filler used in this study was comprehensively characterized to evaluate the physical and chemical properties crucial for its performance in the resin-ceramic composite. Figure 2A shows the size distribution of ZnO/SiO_2_ particles. These particles exhibited a relatively narrow size distribution within a range of ~1.3 to 3.6 μm. The mean particle size was ~2.2 μm. This uniform size distribution is beneficial for achieving consistent dispersion within the resin matrix and ensuring homogeneous properties in the final composite [41,42]. The morphology of the ZnO/SiO_2_ particles was examined using SEM, as shown in Figure 2B,C. Primary particles, ~200 nm in diameter, were observed to form agglomerated, spherical-like secondary particles in the micrometer range. The measured size distribution using image analysis (Figure 2A) represents these secondary particle sizes, with a dominant diameter range of 1 to 4 μm and a peak around 2.1 μm. This is consistent with the overall morphology observed in the low-magnification SEM image (Figure 2B), confirming the formation of flowable agglomerates. Such morphology can contribute to improved rheological properties of the composite suspension by providing effective flowability of the filler within the resin matrix [43,44].

Furthermore, energy-dispersive X-ray spectroscopy (EDS) analysis was performed to evaluate the elemental composition of the ZnO/SiO_2_ ceramic filler. The average weight percentages measured are summarized in Table 2, based on five-point EDS spot measurements. To approximate the composition in terms of oxide phases (ZnO and SiO_2_), the weight percentages were converted into ZnO and SiO_2_ equivalents by considering their respective molar masses. As a result, the filler composition was estimated to consist of approximately ~59.5 wt% ZnO and ~40.5 wt% SiO_2_. It should be noted that these values confirm the presence of both oxides in the filler system and validate the use of the term ‘ZnO/SiO_2_ ceramic filler’ in this work.

**Figure 3:**
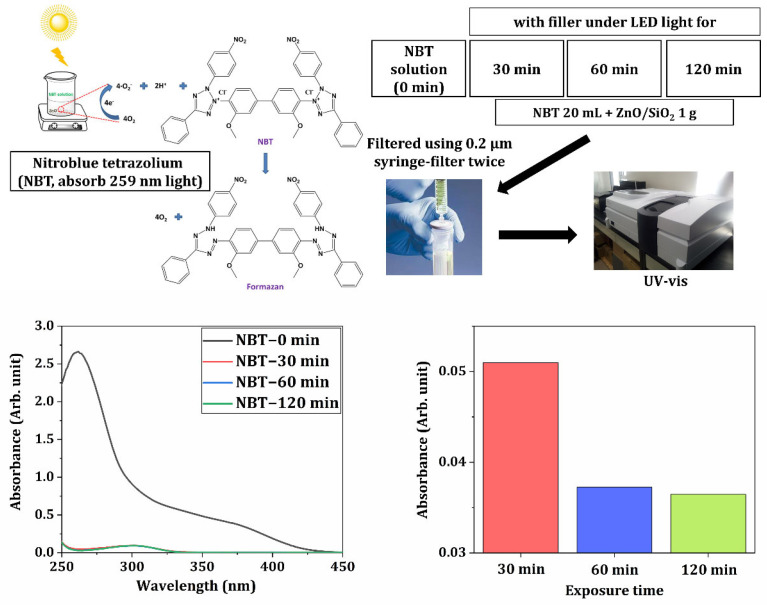
Results of ROS generation by ZnO/SiO_2_ particles using UV-Vis spectroscopy for 30, 60, and 120 min.

X-ray diffraction (XRD) analysis was performed to investigate the crystalline structure of the ZnO/SiO_2_ filler (Figure 2D). Characteristic peaks of the as-received filler could not be observed in the XRD data. This suggests the presence of amorphous silica consistent with the composite nature of the ZnO/SiO_2_ filler. One of the key properties of ZnO for antimicrobial applications is its ability to generate reactive oxygen species (ROS). Figure 3 demonstrates the ROS generation capability of the ZnO/SiO_2_ filler as measured by UV-Vis spectroscopy. Absorbance spectra showed a decrease in the characteristic peak of the NBT indicator over time (30, 60, and 120 min), which demonstrated the production of ROS [45,46]. This confirms that the ZnO/SiO_2_ filler retains its ability to generate ROS, which is a crucial factor for its anticipated antimicrobial activity in the final composite. The combination of appropriate particle size, morphology, crystalline structure, and ROS generation capability makes this ZnO/SiO_2_ filler a promising candidate for incorporation into dental resin composites, potentially improving both mechanical properties and antimicrobial activity.

### 3.2. Rheological Behavior of Resin-Ceramic Composite Suspensions

**Figure 4:**
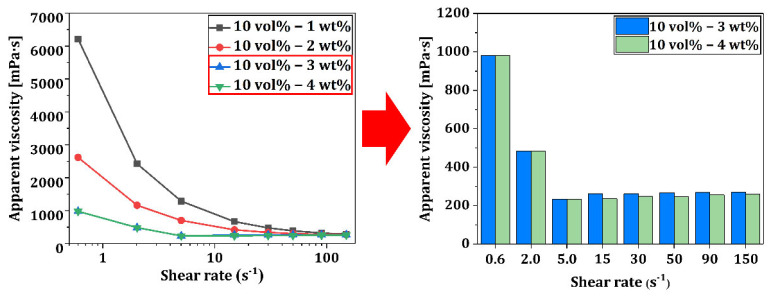
Apparent viscosities as a function of shear rate obtained for resin-ceramic composite suspension with 10 vol% ZnO/SiO_2_ content at 25 °C with different dispersant contents (1, 2, 3, and 4 wt%).

Rheological properties of resin-ceramic composite suspensions are crucial for successful 3D printing as they can affect the flow behavior and printability of the material [47,48]. Figure 4 illustrates apparent viscosities of the resin-ceramic composite suspension containing 10 vol% ZnO/SiO_2_ as a function of shear rate. Apparent viscosities were measured at 25 °C with varying dispersant contents (1, 2, 3, and 4 wt%). Results suggest that all suspensions exhibited shear-thinning behavior. Viscosity decreased as shear rate increased. This non-Newtonian behavior is beneficial for 3D printing applications, as it is a typical characteristic of highly concentrated ceramic suspensions. When applied to vat photopolymerization-related 3D printing, this behavior allows for easy flow of suspension during platform movement, followed by rapid stabilization of suspension [25,49]. The effect of dispersant concentration on viscosity was evident from the graph. As the dispersant content increased from 1 wt% to 3 wt%, viscosity showed a significant reduction across all shear rates. This decrease in viscosity can be attributed to improved dispersion of ZnO/SiO_2_ particles in the resin matrix, reducing particle agglomeration and allowing for better flow. Interestingly, increasing the dispersant content from 3 wt% to 4 wt% did not lead to a further substantial decrease in viscosity. Viscosity curves for 3 wt% and 4 wt% dispersant were very similar, suggesting that 3 wt% dispersant might be the optimal concentration for achieving good dispersion and desirable rheological properties. The lowest viscosity achieved with 3 wt% dispersant is particularly advantageous for vat photopolymerization techniques such as LCD-based 3D printing. Lower viscosity facilitates better recoating of the resin surface between layers, potentially leading to improved print quality and resolution [50,51]. Based on these results, a dispersant concentration of 3 wt% was selected for further experiments, as it provided the optimal balance between good dispersion and low viscosity without unnecessary addition of a dispersant, which could potentially affect the mechanical properties and curing behavior of the final composite.

### 3.3. Photocuring Behavior of Resin-Ceramic Composites

**Figure 5:**
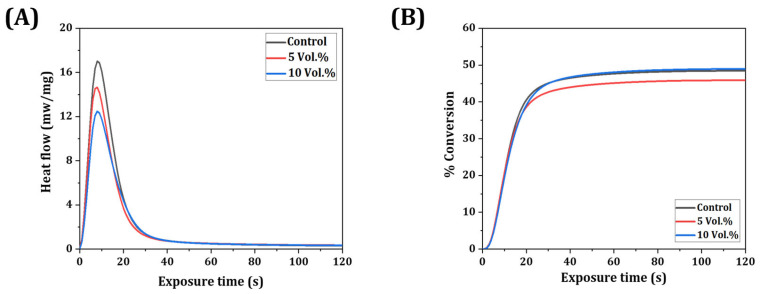
Photo-DSC evaluation results of the photocuring behavior of ZnO/SiO_2_ resin-ceramic composite suspensions with various ceramic contents (0, 5, and 10 vol%) showing (**A**) heat flow and (**B**) percent conversion of polymerization.

Photocuring behavior of ZnO/SiO_2_ resin-ceramic composites was investigated to understand the effect of filler content on the polymerization process. Figure 5 presents the results of photo-DSC analysis for resin-ceramic composites with varying ZnO/SiO_2_ contents (0, 5, and 10 vol%). Figure 5A shows heat flow curves during photopolymerization. All compositions exhibited an initial rapid increase in heat flow upon UV exposure, followed by a gradual decrease as the polymerization reaction progressed. Total Enthalpy (H_t_) decreased with increasing filler content, which could be attributed to light scattering and absorption by ZnO/SiO_2_ particles [52,53]. Figure 5B illustrates the percentage conversion of polymerization over time. All resin-ceramic composite suspensions with varying ZnO/SiO_2_ contents (0, 5, and 10 vol%) showed similar % conversion, which were in the range of ~46% to 49%. Final percent conversion results of all suspensions were not sufficiently high due to the incorporation of UDMA monomer [54,55]. However, the post-curing process was conducted for all samples in a UV-curing chamber to fully cure uncured parts.

**Figure 6:**
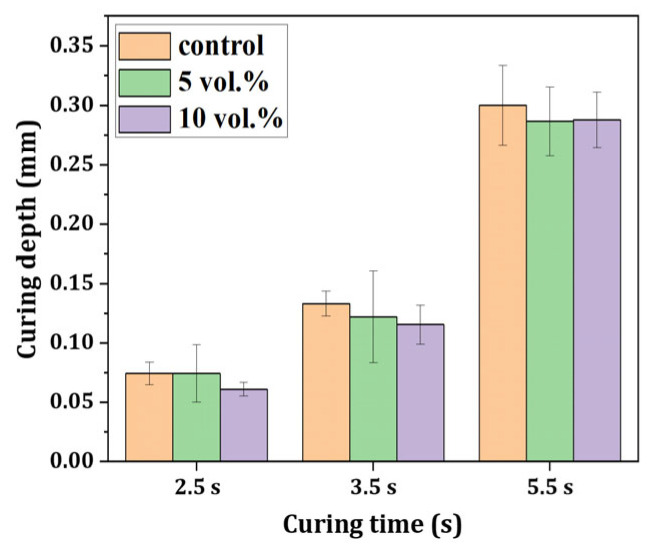
Layer thicknesses of cured ZnO/SiO_2_ resin-ceramic composite with various ceramic contents (0, 5, and 10 vol%) as a function of exposure time for photopolymerization.

Figure 6 demonstrates the effect of exposure time on cure depth for different filler contents. As expected, the cure depth increased with longer exposure time for all compositions. Moreover, cure depth showed a decreasing trend as filler was added. However, there was no statistical difference between suspensions with different filler contents. Furthermore, the observed reduction in polymerization conversion with increasing filler content (Figure 5 and Figure 6) can be attributed to the light scattering and attenuation effects induced by the refractive index mismatch between the resin matrix and the ZnO/SiO_2_ ceramic filler [24,49,56,57,58]. In particular, the higher refractive index of ZnO (~2.0) compared to typical dental resins promotes light scattering during photocuring, thereby limiting light penetration and reducing curing depth and conversion efficiency [57,58]. Thus, a curing time of 2.5 s was set to fully cure a layer thickness of 50 μm in this work. These results highlight the importance of optimizing photocuring parameters based on the specific filler content to achieve the desired balance between curing time, layer thickness, and resolution in the 3D printing process.

### 3.4. Optimization of 3D Printing Parameters

**Figure 7:**
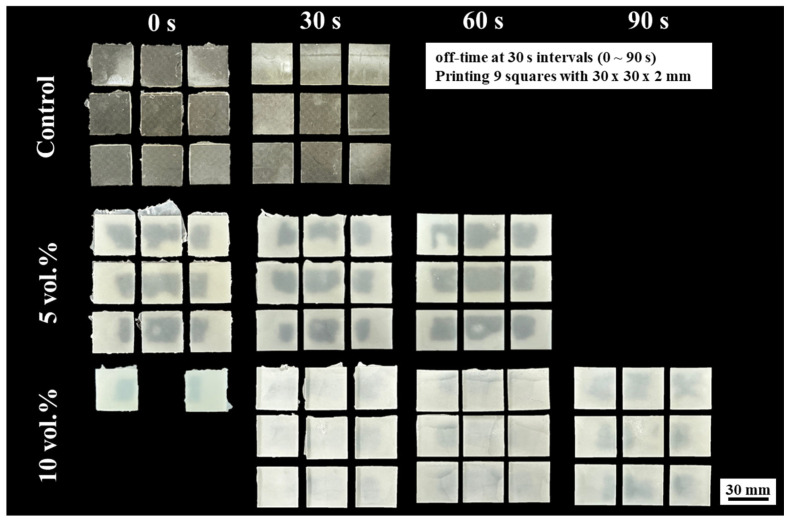
LCD 3D-printed squares with different off-times (0, 30, 60, and 90 s) using ZnO/SiO_2_ resin-ceramic composites with various ceramic contents (0, 5, and 10 vol%).

Optimization of 3D printing parameters is crucial for achieving high-quality prints with desirable properties. In this study, the effect of off-time on the printing quality of ZnO/SiO_2_ resin-ceramic composites with various ceramic contents (0, 5, and 10 vol%) was evaluated. Figure 7 illustrates the results of LCD 3D-printed squares with different off-times (0, 30, 60, and 90 s) for each composition. For the unfilled resin (0 vol% ZnO/SiO_2_), successful prints were achieved with a shorter off-time (30 s), indicating faster recoating and settling of the resin between layers. As the filler content increased to 5 vol% and 10 vol%, longer off-times were required to produce well-defined squares. At 0 s off-time, all compositions showed compromised geometrical accuracy, suboptimal print quality, and incomplete or distorted geometries. This was likely due to insufficient time for the resin to flow and settle between layers, resulting in inadequate recoating and potential adhesion issues. For the ×5 vol% ZnO/SiO_2_ composition, optimal print quality was achieved with a 60 s off-time. Squares printed with this off-time exhibited sharp edges and good dimensional accuracy. Shorter off-time resulted in distorted prints, while longer off-time (90 s) did not show significant improvement in print quality. The 10 vol% ZnO/SiO_2_ composition required the longest off-time (90 s) to produce well-defined squares. This increased off-time requirement can be attributed to the higher viscosity of the suspension with increased filler content that requires more time for proper recoating and settling between layers. These results demonstrate the importance of adjusting the off-time based on the filler content of the resin-ceramic composite. As filler content increases, longer off-times are needed to ensure proper recoating and settling of more viscous suspensions. This optimization is crucial for achieving high-quality prints with good dimensional accuracy and surface finish.

### 3.5. Filler Distribution in Printed ZnO/SiO_2_ Resin-Ceramic Composites

**Figure 8:**
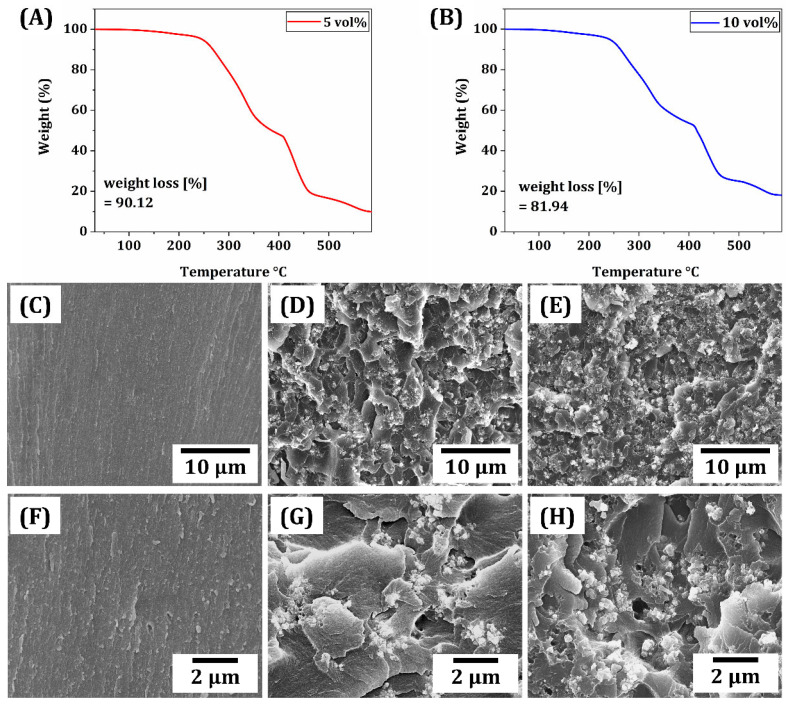
TGA results of resin-ceramic composites with ZnO/SiO_2_ contents of (**A**) 5 vol%, (**B**) 10 vol%, and representative SEM images of fractured surface of resin-ceramic composites with ZnO/SiO_2_ contents of (**C**,**F**) 0 vol%, (**D**,**G**) 5 vol%, and (**E**,**H**) 10 vol%.

A uniform distribution of ZnO/SiO_2_ filler within the resin matrix is crucial for achieving consistent properties throughout printed composites. To evaluate filler distribution, thermogravimetric analysis (TGA) and energy-dispersive X-ray spectroscopy (EDS) mapping were performed for printed samples. Figure 8A,B present TGA results of resin-ceramic composites with ZnO/SiO_2_ contents of 5 vol% and 10 vol%, respectively. Weight loss curves can provide insight into actual filler content and its distribution within printed samples. Results showed distinct differences between the two compositions, with the 10 vol% sample exhibiting a higher residual weight at elevated temperatures than the 5 vol% sample. Furthermore, the result showed a high correlation between the initial ZnO/SiO_2_ content. Theoretically calculated weight percentages of fillers were ~11.5% and ~21.5% for 5 and 10 vol% suspensions, respectively.

To roughly assess the spatial distribution of filler particles, SEM image characterization was conducted. Figure 8C–H shows representative SEM images for the fractured surface of resin-ceramic composites with various ceramic contents (0, 5, and 10 vol%) at different magnifications. The microstructures of resin-ceramic composites were influenced by the content of an inorganic filler in resin composites. As the content increased, visible powders within photopolymerized frameworks increased (Figure 8C–E). The distribution of ceramic fillers appeared relatively uniform, suggesting that filler particles were well-dispersed within the polymer matrix without significant agglomeration or sedimentation. Homogeneous distribution of filler particles is essential for achieving consistent mechanical and antimicrobial properties throughout printed parts [22,59,60,61]. Results from both TGA and SEM images indicated that optimized printing parameters and resin formulation successfully fabricated printed composites with a well-distributed ZnO/SiO_2_ filler. This uniform distribution is expected to contribute to an enhanced performance of printed dental restorations, ensuring consistent mechanical strength and antimicrobial activity across the entire structure.

### 3.6. Mechanical Properties of Printed ZnO/SiO_2_ Resin-Ceramic Composites

**Figure 9:**
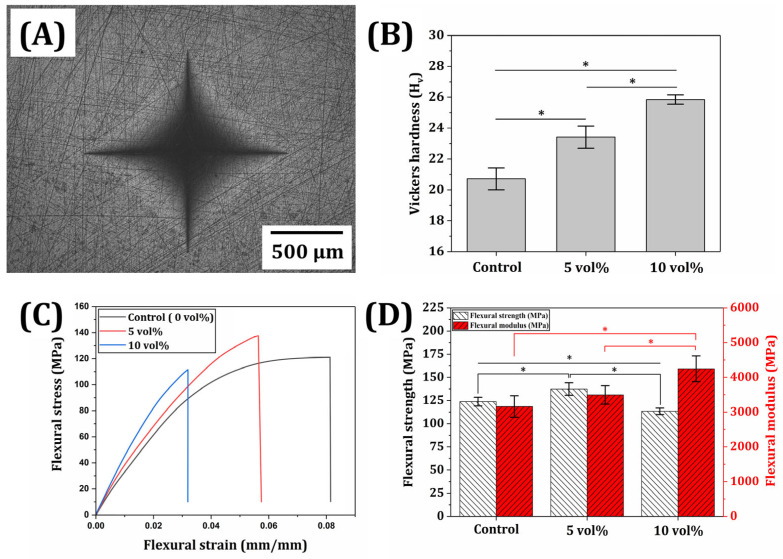
(**A**) Representative image of resin-ceramic composite with ZnO/SiO_2_ content of 10 vol% after Vickers hardness test. (**B**) Vickers hardness values, (**C**) representative three-point flexural stress-strain curve, and (**D**) three-point flexural strengths and modulus of resin-ceramic composites with different ZnO/SiO_2_ contents (0, 5, and 10 vol%).

The incorporation of ZnO/SiO_2_ filler into the resin matrix is expected to influence the mechanical properties of the printed composites. To evaluate these effects, Vickers hardness and three-point flexural tests were conducted for samples with varying filler contents (0, 5, and 10 vol%). Figure 9A,B present results of Vickers hardness tests. A clear trend of increasing hardness with higher filler content was observed. The unfilled resin (0 vol%) exhibited the lowest hardness (20.7 ± 0.7 H_v_), while the composite with 10 vol% ZnO/SiO_2_ ceramic filler showed the highest hardness value (25.9 ± 0.3 H_v_). This enhancement in hardness could be attributed to the reinforcing effect of ceramic filler particles, which could restrict deformation of the polymer matrix under indentation [62]. Note that according to ISO 6507-1 Annex A, the minimum required specimen thickness for a hardness of ~25 H_v_ under a test condition of 294.2 N is approximately ~2.3 mm. Since all printed samples used for Vickers hardness testing had a thickness of 3.5 mm, they fully satisfied the ISO 6507 thickness criterion, avoiding substrate influence during indentation [40].

Figure 9C shows representative strain–stress curves for each composition. As the filler content increased, the slopes of the curves became steeper, indicating a higher stiffness of the composites. Moreover, the strain at fracture decreased with increasing filler content, suggesting that the materials exhibited lower deformability and reduced flexural strain before failure. This behavior implies a transition toward more brittle-like fracture characteristics, despite the polymeric nature of the matrix. Figure 9D quantifies three-point flexural strength and modulus for each composition. As filler contents increased from 0 to 10 vol%, flexural modulus increased from 3161.5 ± 309.4 MPa to 4245.8 ± 371.3 MPa. This increase in modulus was consistent with observed changes in stress–strain curves. It could be attributed to the higher stiffness of the ceramic filler compared to the polymer matrix, which was homogenously distributed within the polymer matrix [63]. As the ZnO/SiO_2_ filler content increased from 0 vol% to 5 vol%, the flexural strength increased from 123.8 ± 4.7 MPa to 37.4 ± 6.8 MPa; however, a higher ZnO/SiO_2_ filler content of 10 vol% resulted in a lower flexural strength (113.4 ± 3.5 MPa). This change in flexural strength is presumably attributed to the dispersion and aggregate of fillers in the resin matrix. More specifically, when proper amounts of fillers (e.g., 5 vol% in this study) are employed, they can be uniformly dispersed in the polymer matrix (cf., Figure 8D). Thus, the filler with much higher strength and stiffness than the resin matrix can effectively withstand flexural (bending) stress, offering enhanced flexural strength, as is often the case with polymer/ceramic composites [58,64]. However, when excessive fillers (e.g., 10 vol% in this study) are used, particles tend to aggregate within the polymer matrix (cf., Figure 8E). These aggregations would not only weaken interfacial bonding to the resin matrix but also cause stress concentration under bending stress, resulting in a decrease in flexural strength [61,63,64,65]. Thus, a number of particles would be detached from the polymer matrix (cf., Figure 8H). However, it should be noted that ZnO/SiO2 filler-incorporated resin composites (5 vol% and 10 vol%) have higher flexural strengths than the requirements (100 MPa) (ISO 4049:2019) for uses as provisional dental materials [38].

### 3.7. Antimicrobial Activities of Printed ZnO/SiO_2_ Resin-Ceramic Composites

**Figure 10:**
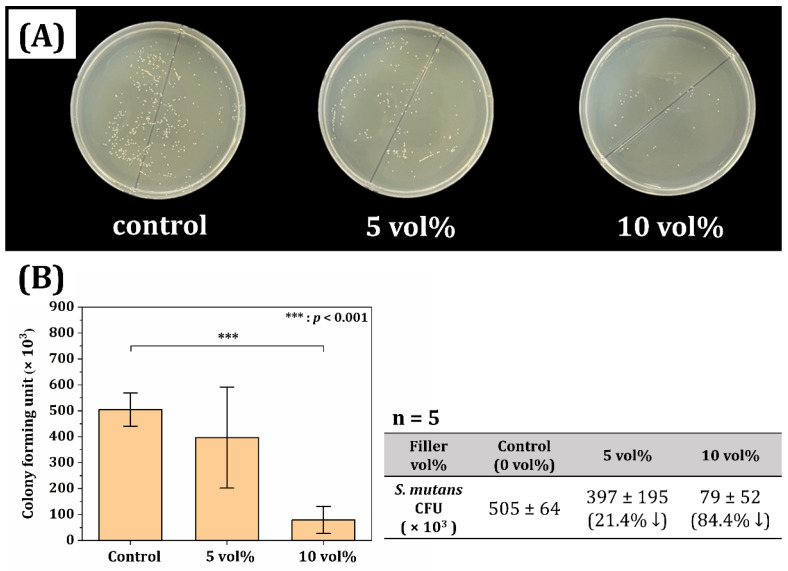
(**A**) Representative image of formed colonies in colony-forming unit (CFU) evaluation and (**B**) results of CFU evaluation of resin-ceramic composites with different ZnO/SiO_2_ contents (0, 5, and 10 vol%).

The incorporation of ZnO/SiO_2_ filler with different contents (0, 5, and 10 vol%) into the resin matrix was primarily motivated by its potential antimicrobial properties. To evaluate the efficacy of this approach, antimicrobial activities of printed composites were assessed using a colony-forming unit (CFU) evaluation method against *S. mutans*, a primary causative agent of dental caries. Figure 10A shows a representative image of the formed colonies resulting from CFU evaluation. The visual difference in colony density between samples with different ZnO/SiO_2_ contents was immediately apparent, suggesting a significant impact of the filler on bacterial growth. Figure 10B shows quantified results of the CFU evaluation for resin-ceramic composites with different ZnO/SiO_2_ contents (0, 5, and 10 vol%). The graph clearly demonstrated a significant reduction in bacterial colonies as the filler content increased. The unfilled resin (0 vol%) showed the highest number of colonies, indicating limited inherent antimicrobial activity. With the addition of 5 vol% ZnO/SiO_2_ filler, there is a substantial decrease (~21.4% decrease with respect to unfilled resin) in the number of colonies, suggesting a notable antimicrobial effect. This reduction became even more evident with 10 vol% filler content, where the number of colonies was drastically reduced compared to both the unfilled resin and the 5 vol% composite (~84.4% decrease with respect to unfilled resin). Our results align with previous findings on the antimicrobial role of ZnO nanoparticles in dental applications [58,66,67], which may be further enhanced through additional experimental strategies such as applying photodynamics [68].

The antimicrobial activity observed could be attributed to several mechanisms associated with ZnO. As demonstrated earlier in ROS generation tests (Figure 3), the ZnO/SiO_2_ filler could produce reactive oxygen species known to damage bacterial cell membranes and inhibit bacterial growth [69,70]. Additionally, the release of Zn^2+^ ions from the filler might have contributed to the antimicrobial effect by interfering with bacterial metabolic processes [70,71]. These results demonstrate the successful incorporation of antimicrobial properties into the 3D printed resin-ceramic composites through the addition of ZnO/SiO_2_ filler. The ability to produce dental restorations with substantial antimicrobial activity represents a significant advancement in the field, potentially leading to improved oral health outcomes for patients receiving these provisional restorations. Therefore, based on the observed changes in mechanical and antimicrobial properties, the null hypothesis was rejected. These findings demonstrate that the addition of ZnO/SiO_2_ ceramic filler significantly influences the functional behavior of the resin composite, demonstrating the importance of compositional optimization for dental applications.

### 3.8. Fabrication of Provisional 3-Unit Bridge Using ZnO/SiO_2_ Resin-Ceramic Composites

**Figure 11:**
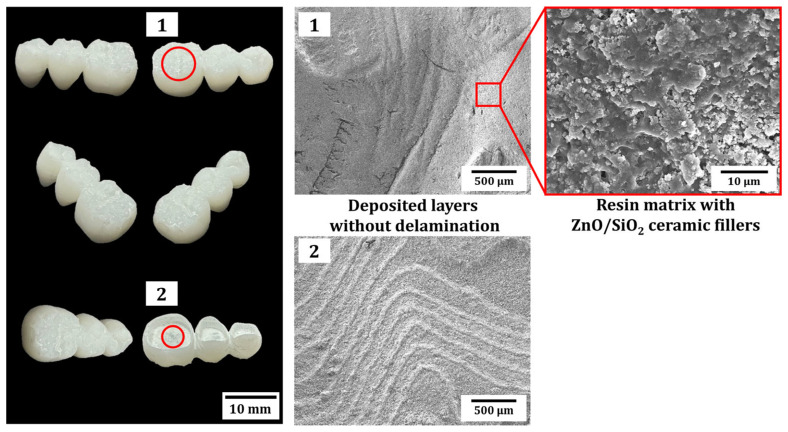
Representative optical image and SEM images of a provisional 3-unit bridge fabricated using resin-ceramic composites with 10 vol% of ZnO/SiO_2_ ceramic fillers.

To determine the practical application of the developed ZnO/SiO_2_ resin-ceramic composite, a provisional 3-unit bridge was fabricated using the optimized LCD 3D printing process. Figure 11 presents a representative image of the printed bridge using the composite with 10 vol% ZnO/SiO_2_ content. The complex geometry of the provisional 3-unit bridge demonstrated the capability of the LCD 3D printing technique to produce intricate dental structures with high precision. The printed bridge exhibited smooth surfaces and well-defined features, without notable failures (cracks, delaminations, and so on), indicating good resolution and accuracy of the printing process. It was worth noting that the ZnO/SiO_2_ filler was exposed to the outer surface, as observed by SEM analysis. This result suggests that ceramic fillers at the surface of the 3-unit bridge had a higher chance for enhanced bacterial inhibition. This is particularly important for dental applications, as it indicates that printed restorations could actively resist bacterial colonization, potentially reducing the risk of secondary caries and other oral infections.

## 4. Conclusions

In this study, an antimicrobial ZnO/SiO_2_ resin-ceramic composite was successfully fabricated and printed using lithography-based liquid crystal display (LCD) 3D printing technology. The curing time of the resin-ceramic composite was optimized through comprehensive analysis of photo-DSC and curing depth measurements. To maintain a high printing accuracy for resin composites with lower flowability due to increased ZnO/SiO_2_ filler content, an off-time parameter was introduced and optimized. This approach allowed for precise control over the printing process, accommodating the changing rheological properties of filled resins. SEM analysis revealed a uniform distribution of filler particles within cured resin specimens. The three-point flexural strength of 5 vol% ZnO/SiO_2_ filler content was the highest, which decreased with higher content of 10 vol%; nevertheless, the strength was higher than the requirement from ISO 4049 (113.4 MPa). However, the stiffness and hardness of the resin-ceramic composite increased with the addition of ZnO/SiO_2_ ceramic filler. Antibacterial testing demonstrated a significant reduction in bacterial count, with up to 84.4% decrease compared to the resin without ZnO/SiO_2_ ceramic filler. A 3-unit provisional bridge containing 10 vol% of ZnO/SiO_2_ ceramic filler was successfully printed using optimized 3D printing conditions, validating the practical applicability of the resin-ceramic composite developed for complex dental structures. This study contributes to the field of digital dentistry by demonstrating an approach to creating functional, antimicrobial dental restorations using additive manufacturing techniques. It opens new possibilities for personalized, high-performance dental prosthetics.

## Figures and Tables

**Table 1:** Compositions of ZnO/SiO_2_-incoptarted resin composites.

**Composite** (**0 vol%**)	ZnO/SiO_2_ filler	UDMA	TEGDMA	HEMA	BYK-2001 (3.0 wt% of filler)	TPO (3.0 wt% of monomer)
Amount (g)	0	5.00	2.00	3.00	0	0.30
Density (g/cc)	2.68	1.11	1.07	1.07	1.03	1.12
vol%	**0**	47.69	19.79	29.68	0	2.84
**Composite** (**5 vol%**)	ZnO/SiO_2_ filler	UDMA	TEGDMA	HEMA	BYK-2001 (3.0 wt% of filler)	TPO (3.0 wt% of monomer)
Amount (g)	1.34	5.00	2.00	3.00	0.04	0.30
Density (g/cc)	2.68	1.11	1.07	1.07	1.03	1.12
vol%	**5.00**	45.12	18.72	28.08	0.40	2.68
**Composite** (**10 vol%**)	ZnO/SiO_2_ filler	UDMA	TEGDMA	HEMA	BYK-2001 (3.0 wt% of filler)	TPO (3.0 wt% of monomer)
Amount (g)	2.84	5.00	2.00	3.00	0.08	0.30
Density (g/cc)	2.68	1.11	1.07	1.07	1.03	1.12
vol%	**10.00**	42.55	17.66	26.49	0.77	2.53

**Table 2:** Elemental and chemical compositions of the ZnO/SiO_2_ ceramic filler characterized by EDS spot analyses.

	Elements (wt%)			Compositions (wt%)	
	Zn	Si	O	ZnO	SiO_2_
ZnO/SiO_2_ ceramic filler	48.5 ± 0.8	18.5 ± 0.5	33.0 ± 0.4	59.5 ± 0.7	40.5 ± 0.7

## Data Availability

The original contributions presented in the study are included in the article, further inquiries can be directed to the corresponding authors.
